# Characterization and comparative analysis of the complete chloroplast genome sequence from *Prunus avium* ‘Summit’

**DOI:** 10.7717/peerj.8210

**Published:** 2019-12-20

**Authors:** Xueqing Zhao, Ming Yan, Yu Ding, Yan Huo, Zhaohe Yuan

**Affiliations:** 1 Co-Innovation Center for Sustainable Forestry in Southern China, Nanjing Forestry University, Nanjing, Jiangsu, China; 2 College of Forestry, Nanjing Forestry University, Nanjing, Jiangsu, China; 3 College of Landscape Architecture, Nanjing Forestry University, Nanjing, Jiangsu, China

**Keywords:** *Prunus avium*, Chloroplast genome, Genome comparison, Phylogenetic analysis, SSR

## Abstract

**Background:**

Sweet cherry (*Prunus avium*) is one of the most popular of the temperate fruits. Previous studies have demonstrated that there were several haplotypes in the chloroplast genome of sweet cherry cultivars. However, none of chloroplast genome of a sweet cherry cultivar were yet released, and the phylogenetic relationships among *Prunus* based on chloroplast genome data were unclear.

**Methods:**

In this study, we assembled and annotated the complete chloroplast genome of a sweet cherry cultivar *P. avium* ‘Summit’ from high-throughput sequencing data. Gene Ontology (GO) terms were assigned to classify the function of the annotated genes. Maximum likelihood (ML) trees were constructed to reveal the phylogenetic relationships within *Prunus* species, using LSC (large single-copy) regions, SSC (small single-copy) regions, IR (inverted repeats) regions, CDS (coding sequences), intergenic regions, and whole cp genome datasets, respectively.

**Results:**

The complete plastid genome was 157, 886 bp in length with a typical quadripartite structure of LSC (85,990 bp) and SSC (19,080 bp) regions, separated by a pair of IR regions (26,408 bp). It contained 131 genes, including 86 protein-coding genes, 37 transfer RNA genes and 8 ribosomal RNA genes. A total of 77 genes were assigned to three major GO categories, including molecular function, cellular component and biological process categories. Comparison with other *Prunus* species showed that *P. avium* ‘Summit’ was quite conserved in gene content and structure. The non-coding regions, *ndhc*-*trnV*, *rps12*-*trnV* and *rpl32*-*trnL* were the most variable sequences between wild Mazzard cherry and ‘Summit’ cherry. A total of 73 simple sequence repeats (SSRs) were identified in ‘Summit’ cherry and most of them were mononucleotide repeats. ML phylogenetic tree within *Prunus* species revealed four clades: *Amygdalus*, *Cerasus*, *Padus*, and *Prunus*. The SSC and IR trees were incongruent with results using other cp data partitions. These data provide valuable genetic resources for future research on sweet cherry and *Prunus* species.

## Introduction

Chloroplast (cp) is generally situated in the cytoplasmic matrix and plays an important role in photosynthesis and fatty acid, starch, and amino acid synthesis (Wicke et al., 2011).  The cp genome size ranges from 100 kb to 200 kb (Daniell et al., 2016). It is a typical quadripartite structure that consists of large single copy (LSC) region, small single copy (SSC) region, and two inverted repeats (IR) regions; It is well known that the cp genome is usually highly conserved in gene structure and content. Several mutations and small structural changes, such as insertions, deletions, reversals, and translocations have been identified in cp genomes. Therefore, the mutational changes in cp genome sequences provide valuable information for phylogenetic, genetic diversity analysis and molecular marker development.

*Prunus* L., a large and diverse genus, comprises more than 400 species, including most of economically important fruit crops as well as many ornamental species. Due to the parallel evolution of morphological traits, and interspecific hybridization, the botanical classification of the *Prunus* L. has long been controversial and complicated. As early as in 1,700, six subgenera within *Prunus* were recognized based on fruit morphology: *Amygdalus* L., *Armeniaca* Mill., *Cerasus* Mill., *Laurocerasus* Duhamel, *Persica* Mill., and *Prunus sensu stricto* (Bouhadida et al., 2007). Afterwards, different opinions, such as a single genus *Prunus* subdivided into seven sections by Bentham and Hooker in 1865, four subgenera within *Prunus* by Koehne in 1911, were also put forward. Currently, the most widely accepted classification of *Prunus* was defined by Rehder in 1940, in which five subgenera *Amygdalus*, *Cerasus*, *Laurocerasus*, *Padus*, and *Prunus* (=*Prundophora*) were divided (Potter, 2011).

As the plastid genome provides more accurate proofs to estimate genetic affinities and phylogenetic relationships, several plastomes of *Prunus* plants have been sequenced and reported, such as *P. persica* (Jansen et al., 2011), *P. yedoensis* (Cho et al., 2016), *P. mume* (Wang, Gao & Gao, 2016), *Amygdalus mira* (Amar et al., 2018), *P. tomentosa* (Chen et al., 2018b), *P. takesimensis* (Cho, Yang & Kim, 2018), *P. mongolica* (Duan et al., 2018), *P. pedunculata* (Duan et al., 2018; Wang et al., 2018a), *P. pseudocerasus* (Feng et al., 2018), *P. serotina* (Luan et al., 2018), *Cerasus humilis* (Mu et al., 2018), *P. cerasoides* (Xu et al., 2018), *P. davidiana* (Zhang et al., 2018), and *P. speciosa* (Sun, Katsuki & Liu, 2019).

Sweet cherry (*Prunus avium* L.) is an important *Prunus* fruit in temperate and sub-tropical regions. Traditional intraspecific and interspecific hybridizations have been carried out in this species for genetic improvement by introducing additional desirable characters (Sansavini & Lugli, 2008; Potter, 2011; Carrasco et al., 2013), and all kinds of cultivars were released to meet the market needs of all over the world. Due to natural multiplication and artificial cultivation for so long time, genetic diversity among cultivars and/or populations were verified by researchers (Frascaria, Santi & Gouyon, 1993; Beaver, Iezzoni & Ramm, 1995; Lacis et al., 2009). Because of the conserved properties of chloroplast DNA (cpDNA), many researchers believed that the chances of detecting intraspecific cpDNA variations were low. However, several haplotypes in sweet cherry populations or cultivars were reported previously (Mohanty, Martín & Aguinagalde, 2001a; Mohanty, Martín & Aguinagalde, 2001b; Panda et al., 2003), which provided a great opportunity to study plastome sequence variation below species level. Recently, the complete chloroplast genome of wild Mazzard cherry (*P. avium*) has been deposited in GenBank (Chen et al., 2018a). However, none of chloroplast genome sequences of a sweet cherry cultivar have yet to be released, which was not conducive to cherry haplotypes variation studies.

In this study, we assembled and analyzed the chloroplast genome of a sweet cherry cultivar ‘Summit’ based on the next-generation sequencing method. Furthermore, we carried out comparative analysis with other *Prunus* species to obtain basic features of cp genomes in *Prunus*. Particularly, general cp genome features and sequence comparison between wild Mazzard cherry and ‘Summit’ were conducted. Phylogenetic trees were also constructed based on the LSC, SSC, IR, CDS (coding sequences), intergenic regions, and the whole chloroplast sequences to study the relationships in genus *Prunus*. The results might benefit the genetics and breeding of cultivated sweet cherries and related *Prunus* species.

## Materials & Methods

### Sampling and DNA extraction

The sample ‘Summit’ tree was grown in Baima Teaching and Research Base of Nanjing Forestry University, Jiangsu Province, China. The voucher specimen was deposited in Nanjing Forestry University Herbarium (NF0000016). Total genomic DNA was extracted from fresh leaves by a CTAB method (Li et al., 2013) with slight modifications. The concentration of DNA was checked by using a Nanodrop ND-2000 spectrometer (Nanodrop Technologies, Wilmington, DE, USA).

### Sequencing, assembly, annotation, and Gene Ontology (GO) analysis

A shortgun DNA library was constructed and the subsequent high-throughput sequencing was carried out on the Illumina HiSeq 2500 Sequencing System (Illumina, CA, USA). Raw paired reads were retrieved, trimmed using Fastp 0.20.0 (Chen et al., 2018b) to obtain clean data. The de novo assembly of the complete cp genome was performed by NOVOPlasty v3.1 program (Dierckxsens, Mardulyn & Smits, 2017). The complete cp genome of *Prunus persica* (HQ336405) was selected as the reference, with *rbcL* as seeds sequence in the analysis. On-line program Geseq (Tillich et al., 2017) was used to annotate the cp genome, and the annotation results were inspected by Geneious 8.0.4 software (Kearse et al., 2012) while modified manually as needed. We deposited the sequence data into GenBank with the accession number MK622380. A physical map of the genome was obtained by using the online tool OGDRAW (Lohse, Drechsel & Bock, 2007). Gene Ontology (GO) annotation was performed by TBtools 1.6 (Chen et al., 2018a) to assign GO terms in our genome data.

### Genome comparison

The cp genome sequence of ‘Summit’ cherry and other 12 additional reported *Prunus* species were compared to analyze the basic features of cp genomes in *Prunus* species. In order to show interspecific variation, five cp genome sequences, *P. avium* (MH756631), *P. persica* (HQ336405), *P. tomentosa* (MF624726), *P. padus* (KP760072), and *Malus prunifolia* (KU851961), were aligned with *P. avium* ‘Summit’ respectively by mVISTA program (Mayor et al., 2000) using Shuffle-LAGAN mode (Dubchak, 2007). The IR expansion and contraction of cp genome among six species was visualized by on-line programme IRscope (Amiryousefi, Hyvönen & Poczai, 2018).

### Simple sequence repeats (SSRs) analysis

Simple sequence repeats (SSRs) in the cp genome of *P. avium* ‘Summit’ were identified using the MsatCommander 0.8.2 program (Faircloth, 2008). The criteria for SSRs identification were 10, 5, 4, 3, 3, 3 repeats units for mono-, di-, tri-, tetra-, penta- and hexa-nucleotides, respectively.

### Phylogenetic analysis

Besides *P. avium* ‘Summit’, an additional 19 *Prunus* species were chosen for phylogenetic analysis, using *M. prunifolia* (KU851961) as an outgroup. The complete cp genome sequences were downloaded from GenBank. Phylogenetic analysis was conducted using the whole genome data, as well as LSC, SSC, IR, CDS, and intergenic regions. The sequences of individual partition regions were aligned using MAFFT v7.308 (Kazutaka & Standley, 2013). A maximum likelihood (ML) tree was implemented in IQ-tree v1.6.8 (Nguyen et al., 2015) under the best-fitting model TVM  + F  + R2. We completed a bootstrap analysis with 1000 replicates. Phylogenetic trees were visualized using the FigTree v1.4.3 software.

## Results

### Characteristics of the chloroplast genome of *P. avium* ‘Summit’

**Figure 1:**
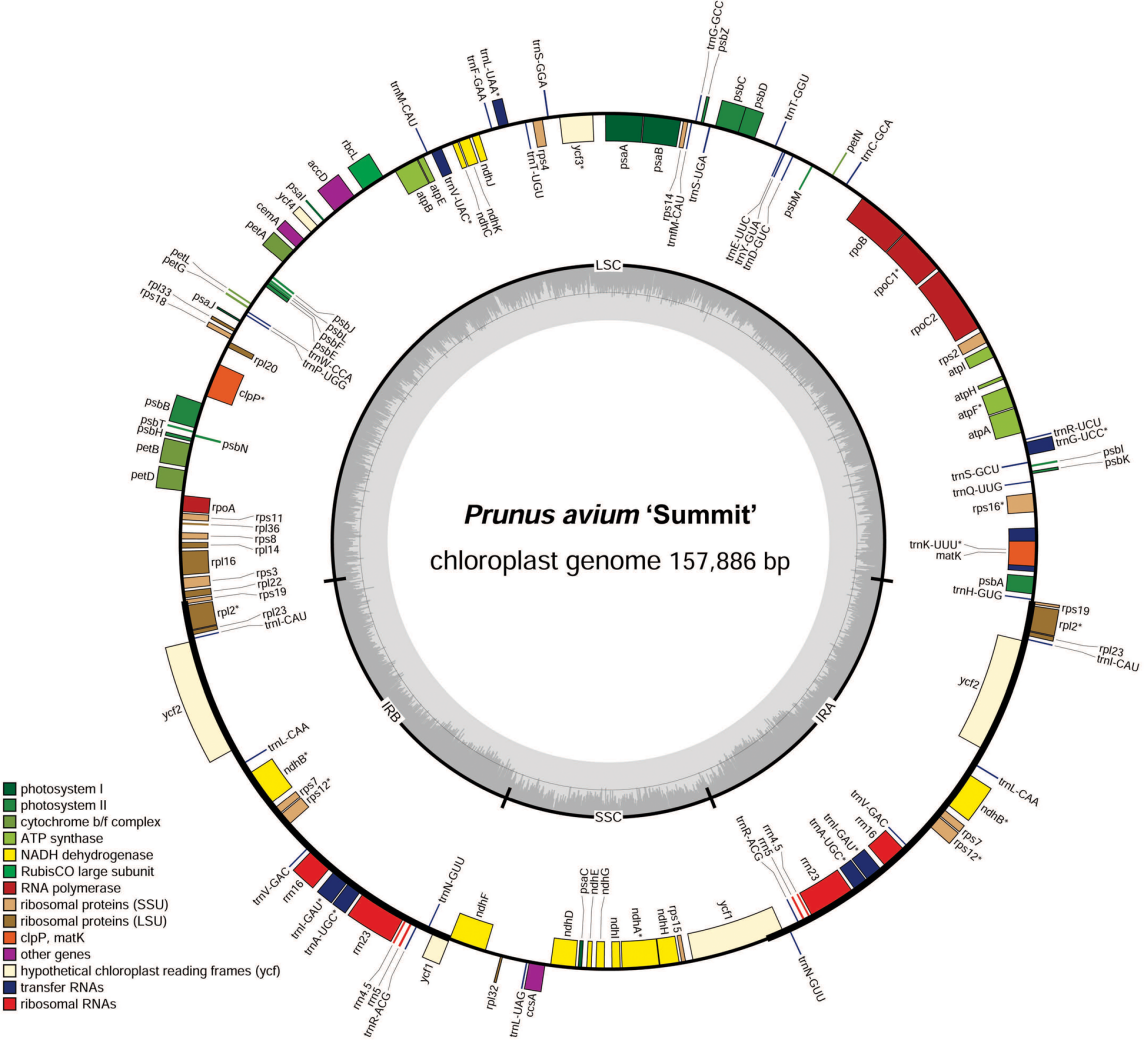
Chloroplast genome map of *P. avium* ‘Summit’. Genes inside the circle are transcribed clockwise, and those outside are transcribed counterclockwise. Genes of different functions are color-coded. The darker gray in the inner circle shows the GC content, while the lighter gray shows the AT content.

In order to facilitate sequence annotation and subsequent analysis accurately, the sequencing low-quality reads were filtered, yielding 8.53 Gb data for *P. avium* ‘Summit’. The clean data in the GenBank SRA archive were deposited with the accession number PRJNA579503. After sequence assembly, a circularized molecule of 157,886 bp cpDNA was obtained. The whole cp genome exhibited a typical quadripartite structure resembling to most of land plants, with a pair of IRs of 26,408 bp separated by a LSC region of 85,990 bp and a SSC region of 19,080 bp (Fig. 1, Table 1). There were 131 functional genes annotated in the cp genome, including 86 protein-coding genes, 37 tRNA genes and 8 rRNA genes. The majority of genes occurred as a single copy, while 17 of them duplicated, including six protein-coding species (*rps7*, *rps12*, *rpl2*, *rpl23*, *ndhB*, and *ycf2*), seven tRNA (*trnS-AGA*, *trnL-UAG,trnN-GUU*, *trnR-ACG*, *trnA-UGC*, *trnI-GAU*, and *trnV-GAC*), and all four rRNA species (*rrn4.5*, *rrn5*, *rrn16*, and *rrn* 23) (Table 2). Additionally, 13 genes, i.e., *trnA-UGC*, *trnG-UCC*, *trnI-GAU*, *trnK-UUU*, *trnL-UAA*, *trnV-UAC*, *rpoC1*, *rps12*, *rps16*, *rpl2*, *atpF*, *ndhA*, and *ndhB*, contained a single intron, while *ycf3* and *clpP* had two introns (Table 2).

**Table 1:** Basic features of cp genomes of reported *Prunus* species.

**Species**	**GenBank Accession No.**	**Size (kb)**	**LCS length (kb)**	**SSC length (kb)**	**IR length (kb)**	**Protein**	**tRNA**	**Gene**	**GC%**	**Reference**
*P. avium* ‘Summit’	MK622380	157.886	85.990	19.080	26.408	86	37	131	36.7	
*P. avium*	MH756631	157.987	85.975	19.121	26.445	82	35	130	35.72	Chen et al. (2018)
*Cerasus humilis*	MF405921	158.084	86.374	19.038	26.336	90	33	131	36.8	Mu et al. (2018)
*P. serotina*	MF374324	158.788	87.289	18.911	26.294	84	37	130	36.6	Luan et al. (2018)
*P. mongolica*	MG602256	158.039	86.173	19.084	26.391	84	37	131	36.8	Duan et al. (2018)
*P. pedunculata*	MG602257	157.851	86.052	19.029	26.385	85	36	131	36.8	Duan et al. (2018), Wang et al. (2018a), Wang et al. (2018b)
*P. pseudocerasus*	KX255667	157.834	86.954	19.084	26.398	86	37	131	36.7	Feng et al. (2018)
*P. takesimensis*	MG754959	157.948	85.959	19.117	26.436	83	37	128	36.7	Cho, Yang & Kim (2018)
*P. davidiana*	MH460864	158.055	86.248	19.047	26.380	86	37	131	36.8	Zhang et al. (2018)
*P. yedoensis*	KP732472	157.786	85.908	19.120	26.379	86	37	131	36.7	Cho et al. (2016)
*P. mume*	NC_023798	157.712	85.861	19.063	26.394	84	37	131	38.9	Wang, Gao & Gao (2016)
*P. cerasoides*	MF621234	157.685	85.792	19.061	26.416	84	37	129	36.7	Xu et al. (2018)
*P. speciosa*	NO Accession No.	157.916	85.927	19.123	26.433	84	37	129	36.7	Sun, Katsuki & Liu (2019)
*P. persica*	HQ336405	157.790	85.968	19.060	26.381	83	37	128	36. 8	Jansen et al. (2011)

**Table 2:** List of genes annotated in the cp genome of *P.avium* ‘Summit’ sequence.

**Function**	**Family**	**Genes**
Self-replication	tRNA genes	*trnA-UGC*^a^(2); *trnC-GCA*; *trnD-GUC*; *trnE-UUC*; *trnF-GAA*; *trnG-GCC*; *trnH-GUG*; *trnG-UCC*^a^; *trnI-GAU*^a^(2); *trnI-CAU* (2); *trnK-UUU*^a^; *trnL-CAA* (2); *trnL-UAA*^a^; *trnL-UAG*; *trnfM-CAU*; *trnM-CAU*; *trnN-GUU* (2);*trnP-UGG*; *trnQ-UUG*; *trnR-ACG* (2); *trnR-UCU*;*trnS-GCU*; *trnS-GGA*; *trnS-UGA*; *trnT-UGU*; *trnT-GGU*; *trnV-GAC* (2); *trnV-UAC*^a^; *trnW-CCA*; *trnY-GUA*
	rRNA genes	*rrn4.5S* (2); *rrn5S* (2); *rrn16S* (2); *rrn23S* (2)
	DNA-dependent RNA polymerase	*rpoA*; *rpoB*; *rpoC1*^a^; *rpoC2*
	Small subunit of ribosome	*rps2*; *rps3*; *rps4*; *rps7* (2); *rps8*; *rps11*; *rps12*^a^(2); *rps14;rps15*; *rps16*^a^; *rps18*; *rps19*
	Large subunit of ribosome	*rpl2*^a^(2); *rpl14*; *rpl16*; *rpl20*; *rpl22*; *rpl23* (2); *rpl32*; *rpl33*; *rpl36*
Photosynthesis	ATP synthase	*atpA*; *atpB*; *atpE*; *atpF*^a^; *atpH*; *atpI*
	Photosystem I	*psaA*; *psaB*; *psaC*; *psaI*; *psaJ*; *ycf3*^b^; *ycf4*
	Photosystem II	*psbA*; *psbB*; *psbC*; *psbD*; *psbE*; *psbF*; *psbH*; *psbI*; *psbJ*; *psbK*; *psbL*; *psbM*; *psbN*; *psbT*; *psbZ*
	Calvin cycle	*rbcL*
	Cytochrome complex	*petA*;*petB*; *petD*; *petG*; *petL*; *petN*
	NADH dehydrogenase	*ndhA*^a^; *ndhB*^a^(2); *ndhC*; *ndhD*; *ndhE*; *ndhF*; *ndhG*; *ndhH*; *ndhI*; *ndhJ*;*ndhK*
Other genes	Others	*ycf1*; *ycf2* (2); *ccsA*; *pbf1*; *clpP*^b^; *cemA*; *accD*; *lhbA*; *matK*

**Notes.**

a Genes containing one intron.

b Genes containing two introns.

**Figure 2:**
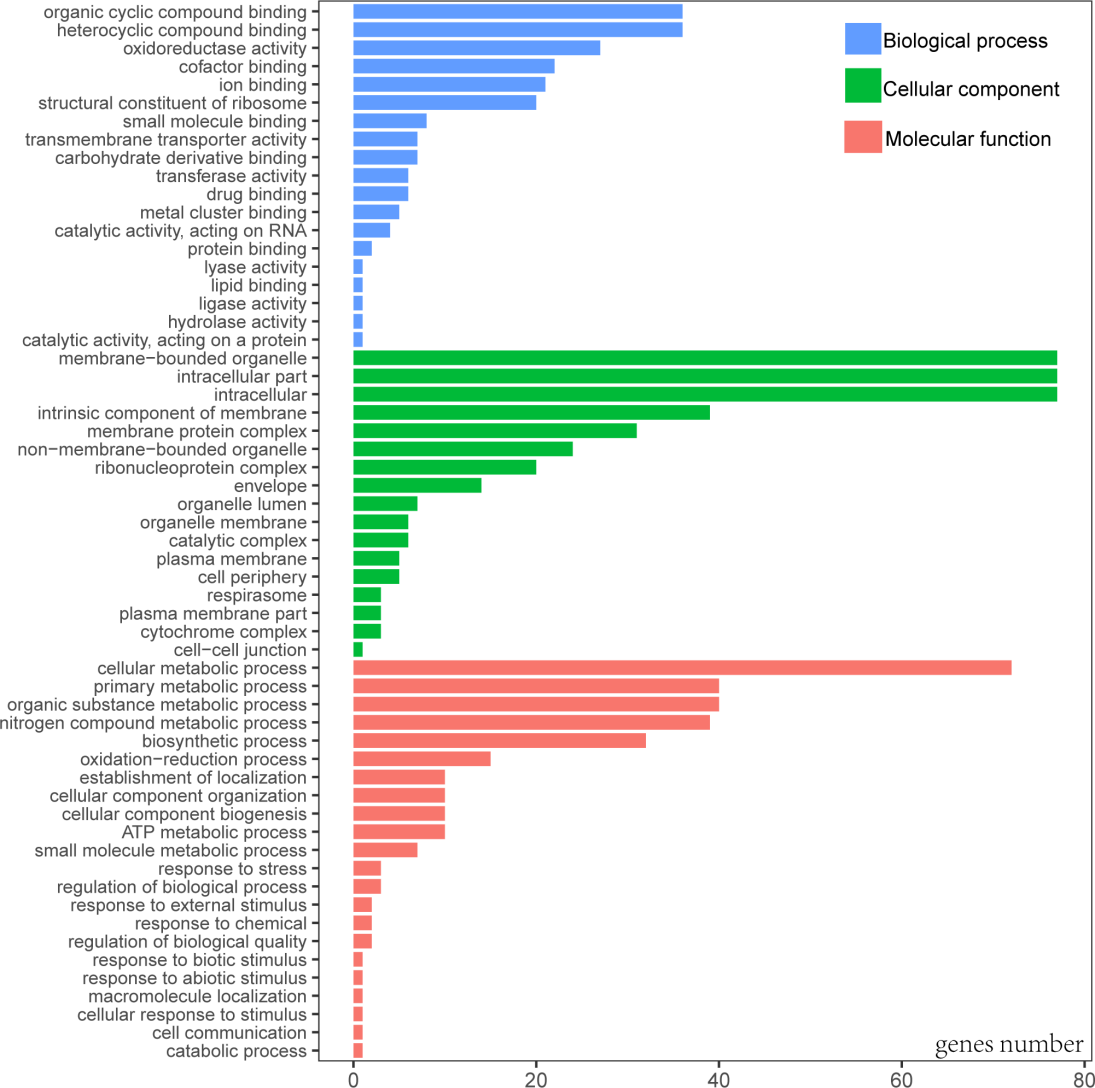
Gene Ontology (GO) annotation of genes from *P. avim* ‘Summit’.

When Gene Ontology (GO) was conducted, only 3 genes (*rps19*, *pbf1*, and *lhbA*) were unable to be annotated. According to the GO result, the most functional groups (15) were identified in *psaA* and *psbA* genes (Table S1). Predicted genes of cp genome were functionally classified according to the three main GO categories including 58 functional groups (Fig. 2, Table S2). Molecular functional categories were strongly represented by terms related to organic cyclic compound binding (GO:0097159), heterocyclic compound binding (GO:1901363), followed by oxidoreductase activity (GO:0016491). The most common assignments in the cellular component category were membrane-bounded organelle (GO:0043227), intracellular (GO:0005622) and intracellular part (GO:0044424). Genes in the biological process category were primarily sorted into the metabolic process and biosynthetic process.

### Comparative analysis of the cp genomes of genus *Prunus*

The complete cp genome sequence of *P. avium* ‘Summit’ was compared to that of reported *Prunus* species. The results (Table 1) showed that sequenced plastid genomes were similar in terms of organization, gene content, gene order, and GC content. From the aspect of genome size, *P. cerasoides* had the smallest cp genome with the smallest LSC region (85,792 bp), while *P. serotina* had the largest cp genome size with the largest LSC, at 87,289 bp (Table 1). The maximum (26,445 bp) and minimum (26,294 bp) length of IR regions were found in *P. avium* and *P. serotina*. No substantial differences were found in the sequence lengths of SSC among the *Prunus* species. The genome size variation can be explained mainly by differences in the length of LSC and IR regions. The gene number in cp genome covered the range of 128 to 131. Compared with other *Prunus* species, the GC content of *P. mume* was the highest (38.9%) (Table 1).

**Figure 3:**
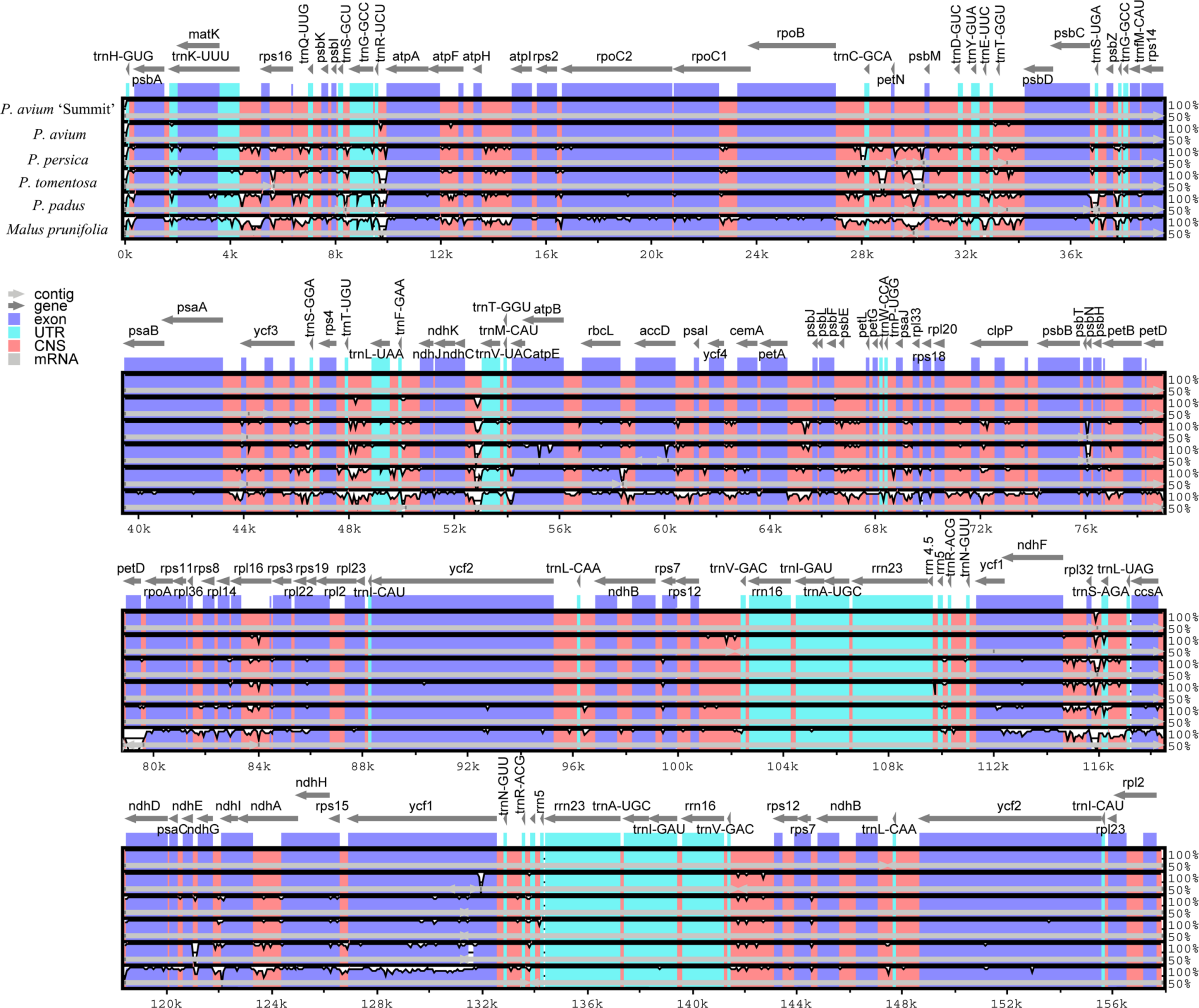
Visualization of genome alignment of the chloroplast genomes of five *Prunus* species and *M. prunifolia* using * P. avium* ‘Summit’ as reference. Y-scale stands for identity from 50 to 100%.

We further calculated sequence similarity for six species of cpDNA using mVISTA by aligning the cp genomes with *P. avium* ‘Summit’ (Fig. 3). Sequence comparison results revealed that the LSC and the SSC regions were more divergent than the IR regions as expected. The highly divergent regions among the six chloroplast genomes mainly occured in the intergenic spacers like *trnH*-*psbA*, *trnK-rps16*, *rps16-trnQ*, *trnS-trnG*, *trnR-atpA*, *atpH-atpI*, *rpoB-trnC*, *trnC-petN*, *petN-psbM*, *trnT-psbD*, *psbC-trnS*, *psbZ-trnG*, *ycf3-trnS*, *trnF-ndhJ*, *ndhC-trnV*, *psbE-petL*, *ndhF-rpl32*, *rpl32-trnL*, and *ndhG-ndhI*. The sequence similarity between Mazzard cherry and ‘Summit’ cherry was relatively high, but several non-coding regions, such as *ndhC*-*trnV*, *rps12*-*trnV* and *rpl32*-*trnL*, exhibited divergence.

### IR expansion and contraction

**Figure 4:**
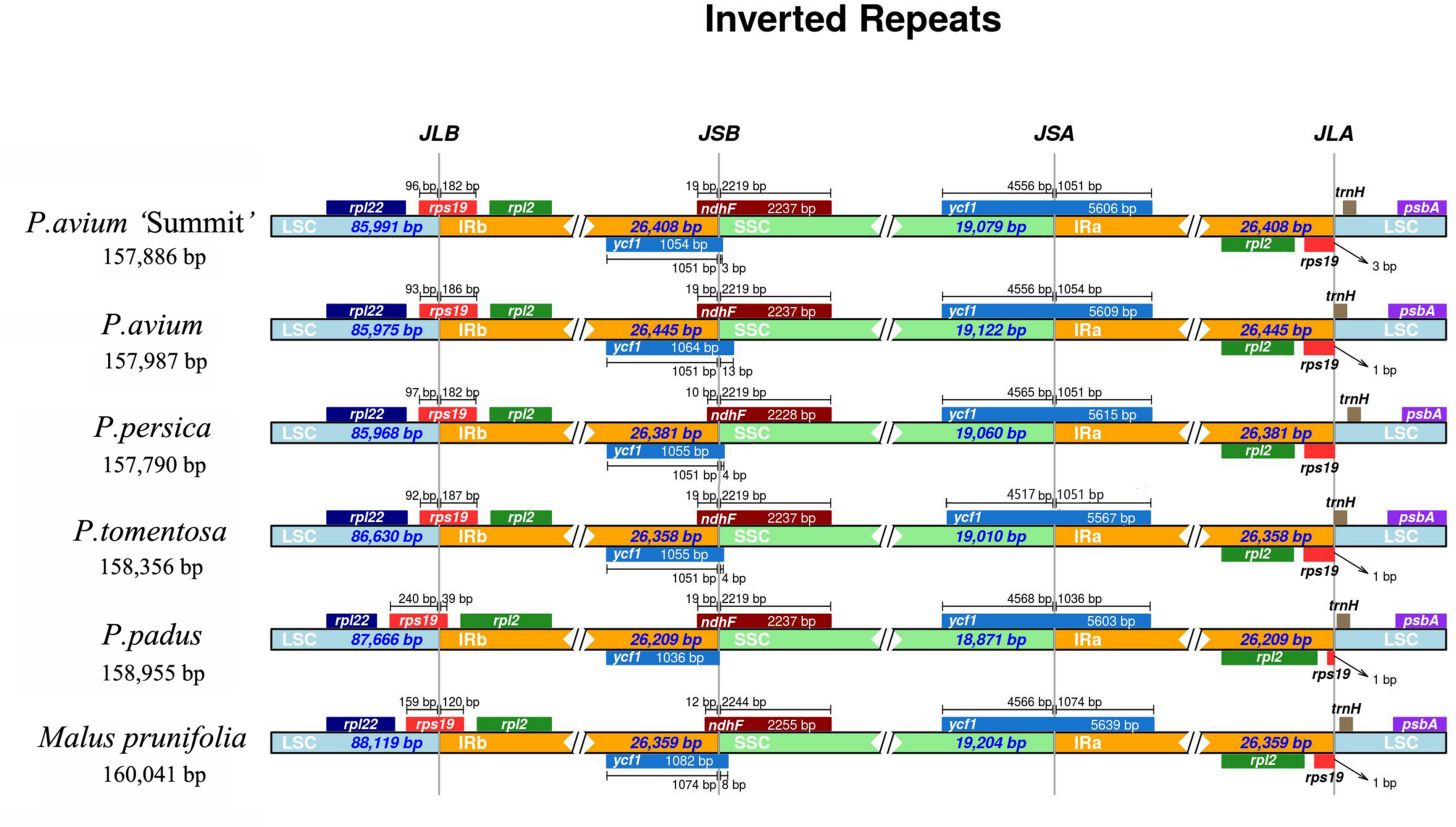
The comparison of IR boundary among 5 *Prunus* species and *M. prunifolia*.

The IR-SSC and IR-LSC boundaries, together with the adjacent genes, among the cp genomes of five *Prunus* species and *M. prunifolia* were aligned. From Fig. 4, *P. avium* ‘Summit’ contained nearly the same IR/SC structure with other congeneric species in which IRb/SC boundaries lay respectively in coding regions of a *rps19* and *ndhF.* For *P. avium* ‘Summit’, *P. avium*, *P. tomentosa* and *P. padus*, it was found to be 19 bp of *ndhF* extension into IRb while a shorter length of 10 bp extension into IRb in *P. persica*. Similarly, the IRb*/* LSC junction was located in the complete *rps19* region in all six species cp genomes and extended into the LSC region by different lengths depending on the species, *P. avium* was 93 bp extension into LSC region while 240 bp in *P. padus.* A truncated *rps19* in IRa region was found, and only 1 bp away from the JLA junction in *P. avium*, *P. tometosa*, *P. padus*, and *M. prunifolia*, while 3 bp in *P. avium* ‘Summit’. Also, the length of *rps19 of P. padus* in IRa region was only 39 bp, which was much shorter than that in other fiver species (180 bp, 186 bp, 183 bp, 187 bp, 120 bp, respectively).

### SSR analysis

In our study, a total of 73 SSRs were identified in the cp genome of *P. avium* ‘Summit’, most of which were detected in the LSC region (Table 3). Among them, 54 (74.0%) were mononucleotide SSRs and most of them belonged to the A/T type, 13 (17.8%) were di-nucleotide SSRs, five (6.8%) were tetra-nucleotide SSRs, one (1.4%) was a penta-nucleotide SSR, there were no tri-nucleotide and hexa-nucleotide SSRs. Only 24 SSRs were located in genes and the others were in the intergenic regions.

**Table 3:** Simple sequence repeats (SSRs) in the *P. avium* ‘Summit’ cp genome.

Repeat unit	Length (bp)	Number of SSRs	Start position
A	10	6	3801(*trnK-UUU*);5663(*rps16*);16438;67241;79696;114707
	11	3	2964;45743;70816
	12	2	27440;65244
	13	3	69114;70112(*rps18*);110074
	14	2	6853;12395(*atpF*)
	15	1	122057
	16	1	130256(*ycf1*)
	18	2	7702;114619
T	10	14	8308;9211(*trnG-UCC*);14546;26359(*rpoB*);28684;44211(*ycf3*);49746; 56081(*atpB*);69383;85316;115469;129653(*ycf1*);130643(*ycf1*)
	11	3	3310(*trnK-UUU*;*matK*);18619(*rpoC2*);122154(*ndhI*)
	12	7	1649;4073(*trnK-UUU*);9601;29278;58797;61300;72286(*clpP*)
	13	3	37286;69086;133792
	14	6	16419;29635;65897;76898(*petB*);84516;123717(*ndhA*)
G	11	1	66458
AT	5	9	6958;13274;19992(*rpoC2*);31097;50125;50370;50381;52952;52968
	6	4	6841;73963;76816(*petB*);115877
AAAT	3	2	5602(*rps16*);72130(*clpP*)
	4	1	1790(*trnK-UUU*)
AATT	3	1	85883
ATTT	3	1	4064(*trnK-UUU*)
AATTT	3	1	32774

### Phylogenetic analysis

**Figure 5:**
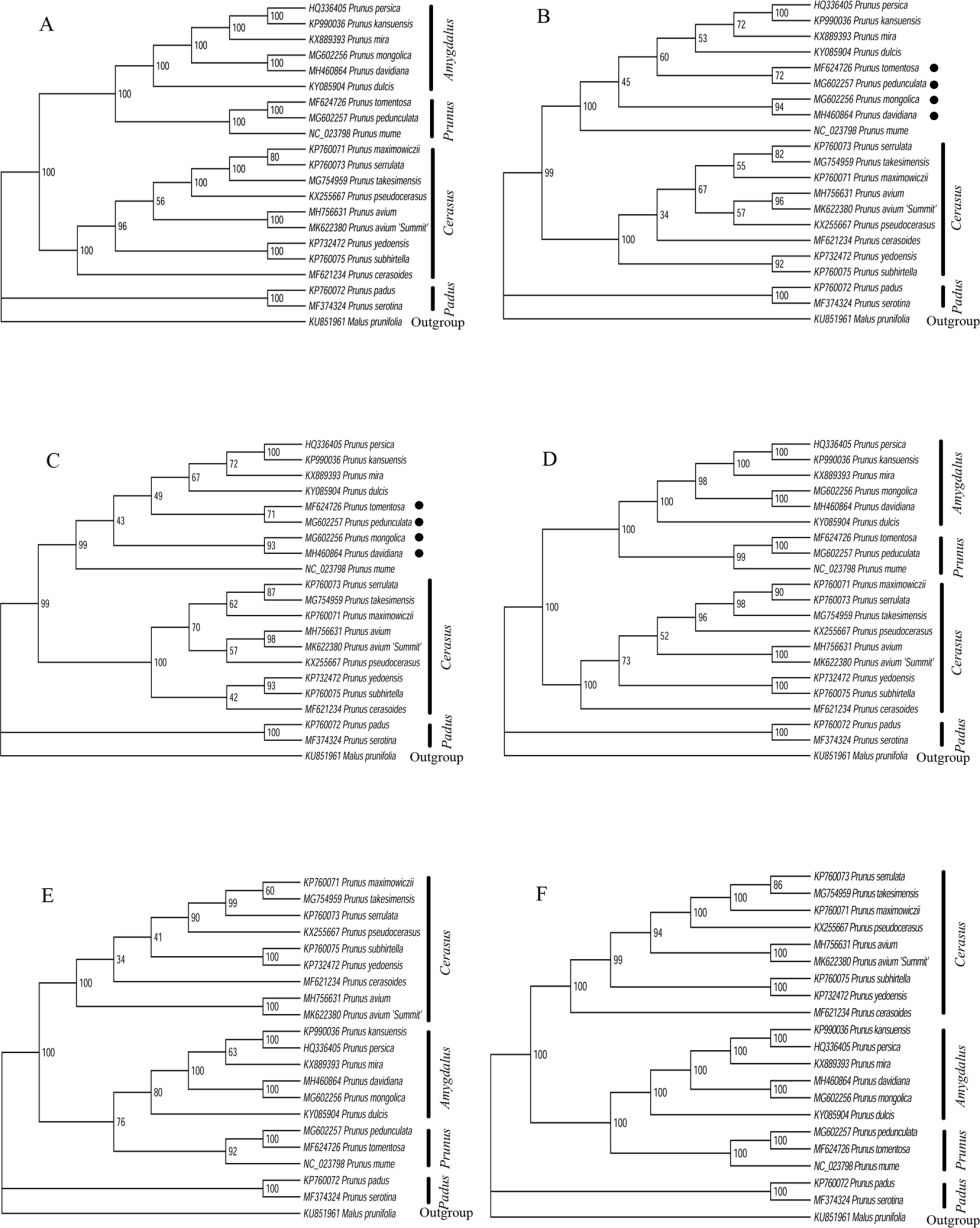
Phylogenetic relationships of 20 *Prunus* species using Maximum likelihood (ML) analysis by six cp genome partition datasets. (A) LSC region. (B) SSC region. (C) IR region. (D) CDS region. (E) intergenic region. (F) whole cp genome.

Six datasets of 20 *Prunus* cp genome sequences were used to build the phylogenetic tree. When the six phylogenetic trees were compared with each other, we found that the topological structures based on LSC region, CDS region, intergenic region and whole cp genome datasets were similar (Fig. 5). The four similar phylogenetic trees demonstrated that the monophyly of the genus *Prunus* was well-supported with a high bootstrap value. Four clades corresponding to subgenus *Amygdalus*, *Cerasus*, *Padus*, and *Prunus* were recovered. The ML trees suggested that the subgenus *Padus*, consisting of *P. padus* and *P. serotine*, was a farther lineage from *Amygdalus* and *Prunus* subgenus than *Cerasus*. The *Amygdalus* clade consisted mainly of peaches and almonds, while the *Prunus* clade consisted of plums, apricots and plum blossom. The subgenus *Cerasus* consisted of tree cherry species including cherry blossoms. Our results confirmed that the *P. avium* ‘Summit’ and Mazzard cherry was a member of *Cerasus* as expected*.* However, there were some inconsistent phylogenetic relationships among species based on the SSC region and IR region datasets. Based on both datasets, the two phylogenetic trees provided a different position of *P. peduculata*, *P. tomentosa*, *P. davidiana*, and *P. mongolica*.

## Disscussion

The cp genome normally has a circular structure, and it is composed of a LSC region, a SSC region and two IR regions. From the results, the genome structure, gene order and GC content of *P. avium* ‘Summit’ were much similar to those reported *Prunus* cp genomes (Cho et al., 2016; Feng et al., 2018; Luan et al., 2018). Through comparative analysis of complete cp genome sequences, much genetic information could be discovered. Our results revealed that the sequence divergence of IR regions was lower than that in LSC and SSC regions, which was also reported in many land plants. In angiosperms cp genomes, the higher divergent intergenic regions, especially the *rpl32-trnL* region, has been used for phylogenetic and evolutionary studies even at the species level (Dong et al., 2012; Zecca et al., 2012; Jara-Arancio, Vidal & Arroyo, 2018). The highly divergent non-coding regions revealed by comparative analysis showed the potentiality for genetic analysis in *Prunus* genus.

Raubeson et al. (2007) pointed out that contraction and expansion at the borders of IR regions were common evolutionary events, and might be the main reason for size diversity of cp genomes. The contraction and expansion result in genes at or near the boundaries such as *rps19* and *ycf1* became truncated as incomplete duplications of the normal copy. In most higher plants of chloroplast genomes, *ycf1* was one of the giant ORFs and it usually spaned the boundary of the IR and SSC regions of the plastid genome (Neubig et al., 2009), but there were some exceptions. Chang et al. (2006) demonstrated that the entire *ycf1* gene in *Phalaenopsis aphrodite* was not across the IR/SSC boundary but within the SSC region. In addition, there were reports on the deletion of *ycf1* gene in IRb/SSC border region in *P. maximowiczii* and *Cerasus humilis* (Mu et al., 2018). The function of *ycf1* gene in the evolution of chloroplast genome requires further investigations.

In this study, two *rps19* genes in the IR/SC boundaries were found. In *Dianthus*, there was one copy of the *rps19* gene at the IRb/SSC junction and the other truncated one at IRA/LSC junction a pseudogene (Raman & Park, 2015). Lu, Li & Qiu (2017) also reported that in three *Cardiocrinum* (Liliaceae) species, the *rps19* gene located in the LSC/IRa boundary apparently lost its protein-coding ability due to partial gene duplication. In our study, pseudogene *rps19* gene located in LSC/IRa boundaries remained to be further elucidated, especially in *P. padus* which a much shorter *rps19* in the LSC/IRa boundary was found.

Further analysis of the cp genomes of wild Mazzard cherry and ‘Summit’ cherry revealed a relatively conserved structure, though there were some variations in both cp genomes. The contraction and expansion of IR regions resulted in minor variation of *rps19* and *ycf1* extension length in IR/SC boundaries. The sequence variations between Mazzard cherry and ‘Summit’ cherry were mostly restricted to the non-coding regions, such as *ndhc*-*trnV*, *rps12*-*trnV* and *rpl32*-*trnL*. Wang et al. (2018a) reported that the intraspecific variation among four peanut varieties cp genomes was also relatively limited. Owing to the conserved properties of cpDNA, cp genome sequence variation was scarcely used below species level. However, the variations in these non-coding regions provides potentials for developing molecular markers in cultivar identification, which has been reported in Fig (Baraket et al., 2008) and olive (Mariotti et al., 2010).

Nuclear SSRs have been recognized as powerful and advantageous genetic markers due to its abundance in genomes, high degree of polymorphism, and co-dominance. A variety of SSR markers have been applied to the analysis of genetic variability, cultivar identification, parentage assessment, and quality control of rootstock in *P. avium* (Guarino et al., 2009; Lacis et al., 2009; Turkoglu et al., 2012; DeRogatis et al., 2013; Ivanovych & Volkov, 2017). Additionally, Molecular markers of cpDNA have been successfully used for assessment of genetic diversity in *P. avium* cultivars and populations. The haplotype diversity in sweet cherry populations or cultivars helped to understand the maternal inheritance of chloroplast genome in sweet cherry (Mohanty, Martín & Aguinagalde, 2001a; Mohanty, Martín & Aguinagalde, 2001b; Panda et al., 2003). Khadivi-Khub et al. (2014) revealed that intraspecific polymorphism was observed by cpSSR primers in *P. avium* and other related *Prunus* species. This intraspecific polymorphism revealed by cpSSR also had conformity with viewpoints of Powell et al. (1995) and Provan et al. (1997). More recently, chloroplast SSRs in *P. salicina* had shown to be highly useful markers for phylogenetic studies in *Prunus* genus (Ohta, Nishitani & Yamamoto, 2005). Furthermore, Turkec, Sayar & Heinze (2006) suggested that cpDNA analysis was a straightforward way to classify cherry cultivars. The cpSSR markers in this study may be further developed for candidate markers to detect genetic diversity among different cultivars and populations in sweet and sour cherry, which will help breeders select parental genotypes aiding to cherry breeding programmes.

Many studies attempted to construct a phylogenetic framework of *Prunus* from different aspects but interspecies relationships within the genus still remained ambiguous. Therefore, the relationships within *Prunus* species need further investigation. Linnaeus divided the *Prunus* into *Amygdalus*, *Padus*, and *Prunus*, and later recognized four genera: *Armeniaca*, *Cerasus*, *Padus* (including *Laurocerasus*) and *Prunus.* In Shi’s analysis, *Amygdalus* and *Prunus* were merged into one subgenus *Prunus*, and three subgenera *Cerasus*, *Prunus* and *Padus* were constructed according to cp regions and nuclear genes data (Shi et al., 2013). Our phylogenetic results based on LSC region, CDS region, intergenic region and whole cp genome datasets recognized four subgenera: *Amygdalus*, *Cerasus*, *Padus*, and *Prunus*, which was in accordance with previous phenotype-based classification of Linnaeus in 1754 and Koehne in 1911 (Lee & Wen, 2001). Without *Laurocerasus* (laurel-cherries) clade in our results may be the limited cp genome datasets and additional cp genome data would be necessary to test the genetic relationship of *Laurocerasus* within *Prunus. P. padus* and *P. serotine* were assigned to subgenus *Padus* which was in line with previous results (Wen et al., 2008; Shi et al., 2013). The position of subgenus *Amygdalus* as sister to *Prunus* was in accordance with the results of Yazbek & Oh (2013) and Yazbek & Al-Zein (2014). However, the monophyletic subgenus *Amygdalus* was contrary to the results of Lee & Wen (2001), who reported *Amygdalus* to be paraphyletic. This difference between mono- and paraphyly of *Amygdalus* may be due to marker or sampling differences. Bortiri, Heuvel & Potter (2006) also deemed that molecular data alone do not support the monophyly of subgenus *Amygdalus*. More molecular data combined morphological data are needed to address this question thoroughly.

Two subgenera *Prunus* and *Amgydalus*, especially members of *P. tomentosa*, *P. pedunculata*, *P. mongolica* and *P. davidiana* were intermixed in SSC and IR trees (Fig. 5), which indicated a close tie between *Prunus* and *Amgydalus*. Previous results also demonstrated that subgenera *Prunus* and *Amgydalus* were more closely related to one another than either to subgenus *Cerasus* (Badenes & Parfitt, 1995; Lersten & Horner, 2000; Lee & Wen, 2001; Wen et al., 2008). According to Rehder’s classification, *P. tomentosa* was classified in Subgenus *Cerasus.* Hybridization studies (Kataoka, Sugiura & Tomana, 1988) and isozyme results (Mowrey & Werner, 1990), together with cp regions and nuclear genes data, demonstrated that it was closer to subgenus *Prunus* rather than to *Cerasus* (Bortiri et al., 2001; Bortiri et al., 2002; Shi et al., 2013). In addition, *P. pedunculata* was traditionally classified as a member of genus *Amygdalus* (Lu & Bartholomew, 2003). However, Yazbek & Oh (2013), Yazbek & Al-Zein (2014), and Duan et al. (2018) suggested that *P. pendunculata* should be excluded from subgenus *Amygdalus*, and recovered in subgenus *Prunus*. *P. mongolica and P. davidiana* were closely related to species of peach (*P. persica*), and previous studies based on molecular and morphological analysis all supported the placement of subgenus *Amygdalus* (Yazbek & Oh, 2013; Yazbek & Al-Zein, 2014). Since previous assertions and results of LSC, CDS, intergenic region and whole cp genome trees in this study did not support the placement of *P. tomentosa*, *P. pedunculata*, *P. mongolica* and *P. davidiana*, thus, we maintained that chloroplast genome datasets, such as LSC, CDS, intergenic region and whole cp genome could be employed to construct phylogenetic inferences in *Prunus*.

Numerous phylogenetic studies based on the cpDNA sequences have been carried out during the past years. The cp genome approaches together with nuclear and phenotypic data can provide complimentary information for genetic analysis in *Prunus*. Our incongruent phylogenetic relationship results among *Prunus* species illustrated that when phylogenetic analysis were conducted, the plastome data partitions should be prepared with meticulous care.

## Conclusions

The study reported the first complete cp genome of a sweet cherry (*P. avium*) cultivar ‘Summit’. Comparison with other *Prunus* species revealed that *P. avium* ‘Summit’ was quite conserved in structure as well as gene content. The cp SSRs and several intergenic regions compared with other *Prunus* species could be selected to develop into valuable DNA markers in further study. The phylogenetic analysis using SSC region and IR region datasets were not in accordance with the results using other cp data partitions and other published phylogenies. LSC, CDS, intergenic region and whole cp genome datasets could be employed to evaluate phylogenetic relationships in *Prunus*.

##  Supplemental Information

10.7717/peerj.8210/supp-1 Table S1 GO annotation terms for each of the genes in the *P. avium* ‘Summit’ cp genome

10.7717/peerj.8210/supp-2 Table S2 GO categories of cp genes including 58 functional groups in the *P. avium* ‘Summit’ cp genome

## Additional Information and Declarations

### Data Availability

The following information was supplied regarding data availability: The raw data is available at NCBI: MK622380, and Genbank SRA: PRJNA579503.
